# Chloride Corrosion Process of Concrete with Different Water–Binder Ratios under Variable Temperature Drying–Wetting Cycles

**DOI:** 10.3390/ma17102263

**Published:** 2024-05-11

**Authors:** Lei Wang, Chunhong Chen, Ronggui Liu, Pinghua Zhu, Hui Liu, Hongwei Jiang, Jiang Yu

**Affiliations:** 1 School of Urban Construction, Changzhou University, Changzhou 213164, China; cczuwl@163.com (L.W.); zph@cczu.edu.cn (P.Z.); liuhui@cczu.edu.cn (H.L.); jianghongwei@cczu.edu.cn (H.J.); cczuyi@163.com (J.Y.); 2 Faculty of Civil Engineering and Mechanics, Jiangsu University, Zhenjiang 212013, China; 3 State Key Laboratory of Silicate Materials for Architectures, Wuhan University of Technology, Wuhan 430070, China

**Keywords:** water–binder ratio, variable temperature, drying–wetting cycles, mechanical properties, free chloride content, erosion depth

## Abstract

In this paper, four water–binder ratios (w/b) of 0.29, 0.33, 0.39, and 0.46 were designed. A variable test temperature was implemented in the drying–wetting cycle test according to the temperature fluctuations in the actual service environment, and the constant temperature test was established as the control group. The mechanical properties and chloride corrosion resistance of concrete with different w/b ratios under variable temperature drying–wetting cycles, as well as the microstructure changes, phase composition, and damage mechanism inside the concrete, were investigated. The results showed that the mechanical properties of concrete increased first and then decreased with drying–wetting cycles increasing, whereas the chloride corrosion resistance continued to decline. A higher w/b exacerbated the deterioration of the concrete performance. A higher w/b increased the porosity, chloride diffusion depth, and chloride content, thus reducing the resistance of chloride corrosion. Compared with w/b = 0.29, the compressive strength, splitting tensile strength, mass, and relative dynamic elasticity modulus of w/b = 0.46 exposed to 60 drying–wetting cycles decreased by 54.50%, 52.44%, 0.96%, and 6.50%, respectively, while the porosity, peak chloride content, and erosion depth increased by 45.12%, 70.45%, and 45.00%. Compared with the drying–wetting cycle with a constant temperature, the cumulative damage caused by the drying–wetting cycle with a variable temperature was greater, resulting in more severe deterioration of concrete performance. The increase in the test temperature significantly accelerated the diffusion rate, penetration depth, and chemical binding capacity of chloride ions. After 60 drying–wetting cycles, the peak chlorine content and erosion depth of w/b = 0.46 under variable temperature cycles were 15.38% and 10.32% higher than those under a constant temperature, while the compressive strength, splitting tensile strength, mass, and relative dynamic elastic modulus were reduced by 7.76%, 14.81%, 0.33%, and 2.40%, respectively. Microscopic analysis confirmed that higher w/b and variable temperature cycles accelerated the decay of mechanical properties and the decline of chloride corrosion resistance. According to the numerical fitting analysis, the w/b should be 0.29~0.39 under the condition that the mechanical properties and chloride corrosion resistance of concrete are met.

## 1. Introduction

Chloride corrosion is one of the main causes of durability problems such as corrosion of reinforcing steel, cracking, and spalling of the protective layer of the components in concrete [1,2,3]. This effect is particularly pronounced in environments with drying–wetting cycles (e.g., splash zones and tidal ranges). In chloride salt environments, the presence of chloride ion concentration gradients and pore fluid saturation gradients within the concrete in the drying–wetting alternating zones results in faster chloride transport and diffusion within concrete. As a result, the drying–wetting alternating zones are often the most severely corroded reinforcing steel in concrete structures, which can lead to concrete structures prematurely entering the overhaul stage or ending their service life in advance [4,5].

Compared with long-term immersion, drying–wetting alternation exacerbates the internal structural damage of concrete. In the drying–wetting cycles, the internal concrete shrinks by drying and expands by wetting and absorbing water. Moreover, in the wetting stage, chloride ions are involved in the chemical reaction to generate expansive salts; in the drying stage, water evaporates, the concentration of salt solution in the pores increases, and salt crystals precipitate [6,7,8]. The pressure on the pore wall accumulates, prompting the pore structure to crack and develop continuously, ultimately leading to a decline in the structural performance of the concrete [9].

The deterioration of concrete properties and reduction in structural durability due to drying–wetting cycles are hot issues of great concern [10]. Currently, most of the research on the performance of concrete in drying–wetting cycle environments is based on drying–wetting cycle tests. However, there is no uniform standard for the drying–wetting cycle test, and the results of different drying–wetting cycle test methods cannot be compared quantitatively. At the same time, the deterioration indexes of concrete performance under drying–wetting cycle conditions are not evaluated in the same way.

Numerous scholars believe that the water–binder ratio (w/b) is an important index of the chloride resistance of concrete, and a lot of research has been carried out on this. Ye et al. [11] designed three mortars with different w/b ratios (0.42, 0.47, and 0.52) and concluded that with w/b increasing, the chloride content in the deep depth tended to increase, as well as the depth of convection zone. This may be attributed to the coarser pore structure in mortar with a larger w/b, which enlarged the porosity and pore size, allowing for more extensive chloride ion penetration. Sun et al. [12] concluded that as w/b increased from 0.26 to 0.30, the apparent diffusion coefficient of chloride ions increased from 10% to 25%. It can be seen that the larger w/b created more channels for chloride ions to diffuse in concrete. Wang et al. [13] obtained that the chloride diffusion coefficient of concrete with a larger w/b was higher, and the chloride diffusion coefficient was more susceptible to temperature. For the four w/b ratios (0.45, 0.50, 0.55, and 0.60) at the same temperature, the chloride diffusion coefficient increased by 1.1~1.3 times for every 0.05 increase in w/b. Zhou et al. [14] designed the drying–wetting cycle test with three w/b ratios (0.35, 0.45, and 0.55) and found that the chloride binding capacity of concrete with smaller w/b was stronger, and the depth of the convection zone and peak chloride concentration decreased with the decrease in w/b. Liu et al. [15] showed that with w/b decreasing, the chloride binding capacity increased, which was due to the increase in the specific area of pores caused by the improvement of the microstructure, which enhanced the physical adsorption of chloride ions. However, some results on concrete exposed to a chloride environment (w/b > 0.4) showed that the chloride binding capacity increased with the increase in w/b [16,17,18]. They agreed that the increase in w/b increased the amount of larger micropores, thereby allowing more external chloride ions to penetrate into the concrete. In addition, Chang et al. [19] reported that the effect of w/b in the range of 0.3~0.7 on chloride binding capacity was insignificant. A similar trend was also obtained in reference [20]. The above studies have shown that the w/b has a significant effect on chloride resistance of concrete. However, researchers have different views on the effect of w/b on the chloride corrosion resistance of concrete, and further systematic research is needed.

In previous studies on the chloride transport mechanism of concrete in an unsaturated state considering the effect of temperature, numerous scholars only considered the effect of the average temperature and ignored the effect of seasonal temperature cycling. The repeated day–night variations are equivalent to placing concrete in a temperature-cycling environment. Therefore, it is more in line with the actual working conditions to consider the chloride ion migration mechanism of concrete under drying–wetting cycles at different temperatures. Boddy et al. [21] concluded that chloride ion corrosion rate was positively correlated with ambient temperature and established a functional relationship between chloride diffusion coefficient and temperature, which could more accurately predict the service life of reinforced concrete. Nguyen et al. [22] and Dousti et al. [23] studied the effects of different exposure temperatures (22~50 °C) on chloride ion diffusion under a drying–wetting cycle and found that chloride penetration depth and chloride diffusion coefficient increased with increasing temperature. Alkhaja et al. [24] exposed concrete specimens to 5% NaCl solution with temperatures of 20 °C and 45 °C for 180 days. The results show that an increase in solution temperature significantly increased the chloride corrosion in ordinary concrete and high-strength concrete, especially at the depth of 45 mm. Oh et al. [25] indicated that different temperature exposure conditions lead to different profiles of chloride ion penetration. The temperature affects greatly the chloride penetration profiles in concrete. The higher the temperature, the greater the penetration of chloride. Song et al. [26] reported that exposure in tropical areas results in higher surface chloride content in concrete jetty structures with a similar level of chloride diffusion coefficient, mainly due to an increased temperature. Matsumura et al. [27] designed three w/b ratios (0.4, 0.5, and 0.6) and immersed concrete specimens in 10% NaCl solution at five temperature levels (25, 45, 65, 80, and 90 °C). It was found that concrete with larger w/b had a larger diffusion coefficient. Based on test results, the diffusion coefficient evaluation equation considering temperature and w/b was proposed. So et al. [28] immersed concrete specimens (w/b = 0.4, 0.5, and 0.6) in 3.5% NaCl solution and designed four temperature levels (20, 40, 65, and 90 °C) to investigate the chloride penetration depth and chloride apparent diffusion coefficient of concrete. The results showed that the chloride penetration depth and chloride apparent diffusion coefficient of concrete increased significantly with the increase in the temperature level, and the concrete with lower w/b had a higher temperature dependency. However, there are few studies on w/b under a variable temperature drying–wetting cycle, and it is of great practical value to study the effect of w/b on chloride corrosion resistance of concrete under a variable temperature drying–wetting cycle.

In this paper, the mechanical properties and chloride corrosion resistance of concrete with different w/b ratios under variable temperature drying–wetting cycles, as well as the microstructure changes and damage mechanism inside the concrete, were studied. A variable test temperature was implemented in the drying–wetting cycle test according to the temperature fluctuations in the actual service environment. In this paper, the compressive strength, splitting tensile strength, mass loss rate, relative dynamic elasticity modulus (RDEM), porosity, free chloride content, and chloride ion erosion depth were used to evaluate the damage of concrete’s macroscopic properties. Additionally, the microstructure and phase composition of concrete before and after drying–wetting cycles were analyzed by scanning electron microscopy (SEM) and X-ray diffraction (XRD). It is imperative to investigate the performance degradation law and chloride ion transport mechanism of concrete under variable temperature drying–wetting cycles, which is of great engineering significance to improve the chloride corrosion resistance of concrete under severe service environments.

## 2. Experimental Program

### 2.1. Raw Materials

**Figure 1:**
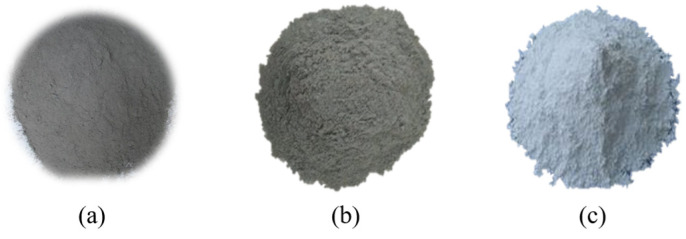
(**a**) Cement, (**b**) fly ash, and (**c**) silica fume.

In this experiment, the cement was P.O 42.5 ordinary Portland cement, and the cementitious materials were fly ash and silica fume. Cement, fly ash, and silica fume are shown in Figure 1, and their chemical compositions are shown in Table 1. Natural coarse aggregate (NCA) is granite gravel with a particle size of 5–20 mm. Natural fine aggregate (NFA) is grade II river sand with a fineness modulus of 2.47. The main properties of the aggregate are shown in Table 2. Superplasticizer polycarboxylate (SP) with a water reduction rate of 25% was used to adjust the workability of concrete.

### 2.2. Mix Proportion and Specimen Preparation

In this study, four w/b ratios of 0.29, 0.33, 0.39, and 0.46 were used to make concrete. The strength grades of concrete with w/b ratios of 0.29, 033, 0.39, and 0.46 were C60, C50, C40, and C30, respectively, and the 28 d compressive strengths were 60.42, 51.36, 42.3, and 34.52 MPa, respectively. Their proportions are presented in Table 3. The full calculation method was adopted in the mixed proportion design [29]. Two-stage mixing method was used for concrete mixing [30]. The cubic specimens with a size of 100 mm × 100 mm × 100 mm were prepared for the compressive strength and splitting tensile strength test of concrete. The cuboid specimens with a size of 100 mm × 100 mm × 400 mm were prepared for the mass loss rate, RDEM, free chloride ion content, and chloride ion erosion depth test of concrete. The specimens were demolded after casting for 24 h, and then they were placed in a standard curing room with a temperature of 20 ± 2 °C and a relative humidity of 95 ± 3% for 28 days.

### 2.3. Exposure Condition and Design of the Drying–Wetting Cycle System

In this study, the actual drying–wetting cycle environment under a coastal concrete tidal zone in Zhoushan City, Zhejiang Province, was simulated. According to the monthly average temperatures in the coastal area of Zhoushan [31] (Table 4), the test temperatures corresponding to different months under the indoor accelerated environment were calculated by Nernst–Einstein Equation (1) [32]. The converted test temperatures are shown in Table 4, and the water temperatures of each drying–wetting cycle were successively used as the corresponding test temperatures for each month. The water temperatures of the first 12 drying–wetting cycles are shown in Table 5. The water temperature of the 13th drying–wetting cycle was the same as the test temperature corresponding to the 1st month, and so on. The concrete with a w/b of 0.46 was used as the control group, and the test environment had a constant water temperature (22 °C) and drying temperature (60 °C).

**Figure 2:**
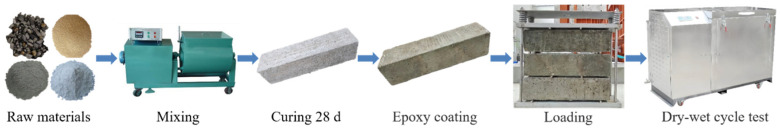
Experimental process chart.

The five sides of the specimens were sealed with epoxy resin, and only one side was left to ensure that the chloride ion was one-dimensional diffusion. A four-point bending loading device [33] was used to apply an ultimate tensile stress of 35% to the concrete specimens by adjusting the length of the spring, so that the concrete specimens could be subjected to a drying–wetting cycle test under continuous loading. Then the loaded specimens were put into the drying–wetting cycle test box. The experimental process is illustrated in Figure 2.

The drying–wetting cycle was wetting followed by drying. The specimens were soaked in 5% NaCl solution for 23 h, then cooled in air for 1 h, then dried at 60 °C for 23 h, and finally cooled in air for 1 h. Two days was a drying–wetting cycle, and a total of 60 drying–wetting cycles were completed. The compressive strength, splitting tensile strength, mass loss rate, RDEM, free chloride content, and chloride ion erosion depth of concrete were tested every 12 drying–wetting cycles.

(1) $$D_{CL}=D_{0}\frac{T}{T_{0}}e^{q(\frac{1}{T_{0}}-\frac{1}{T})}$$

where *D_CL_* and *D*_0_ are chloride diffusion coefficients at temperature *T* and *T*_0_ (K), respectively; *q* is the activation coefficient, which is related to the w/b. When the w/b ratios were 0.33, 0.36, and 0.40, *q* = 6100 K, 6050 K, and 6000 K. For the convenience of the test, the *q* of the concrete with three w/b ratios was 6000 K.

### 2.4. Test Methods

#### 2.4.1. Mechanical Properties Test

The main physical properties of the aggregate were measured according to Chinese standard GB/T 14685-2022 [34]. The compressive strength and splitting tensile strength of concrete were tested according to Chinese standard GB/T 50081-2019 [35]. The cubic specimens with a size of 100 mm × 100 mm × 100 mm were prepared for the compressive strength and splitting tensile strength test of concrete. Three specimens were tested in each group and averaged. If the value in the group exceeded 15% of the average value, it was discarded. The compressive strength and splitting tensile strength were calculated separately by Equations (2) and (3), respectively. The mass loss rate and RDEM of concrete were tested based on Chinese standard GB/T 50082-2009 [36]. The cuboid specimens with a size of 100 mm × 100 mm × 400 mm were prepared for the mass loss rate and RDEM. The mass loss rate and RDME were tested every 12 drying–wetting cycles, and the calculation formulas are shown in Equations (4) and (5), respectively.

(2) $$f_{c}=0.95\frac{F}{A}$$

where *f_c_* is the compressive strength of the non-standard specimen, *F* is the failure load, *A* is the area of the specimen under compression, and 0.95 is the conversion coefficient for compressive strength between non-standard and standard specimens.

(3) $$f_{t}=0.85\frac{2F}{\pi A}$$

where *f_t_* is the splitting tensile strength of the non-standard specimen, *F* is the failure load, *A* is the area of the specimen under splitting, and 0.85 is the conversion coefficient for splitting tensile strength between non-standard and standard specimens.

(4) $$M_{n}=\frac{m_{n}-m_{0}}{m_{0}}\times 100\%$$

where *M_n_* is the mass loss rate of concrete after drying–wetting n cycles, *m_n_* is the mass of concrete after drying–wetting n cycles, and *m*_0_ is the mass of concrete before drying–wetting cycles.

(5) $$E_{rd}=\frac{f_{n}^{2}}{f_{0}^{2}}$$

where *E_rd_* is the RDEM of concrete after drying–wetting n cycles, *m_n_* is the transverse fundamental frequency of concrete after drying–wetting n cycles, and *m*_0_ is the transverse fundamental frequency of concrete before drying–wetting cycles.

#### 2.4.2. Porosity Test

The 100 mm × 100 mm × 100 mm specimens were vacuum saturated, wiped, and weighed as M_0_. Then the specimens were dried to a constant weight in an oven at 105 °C, weighed, and recorded as M_1_. The corresponding porosity of concrete was calculated according to Equation (6). ASTM C642-2013 [37] was used to evaluate the porosity of concrete after 0, 12, 24, 36, 48, and 60 drying–wetting cycles.

(6) $$P=(V_{w}/V_{c})\times 100\%=\left\{\left[\left(M_{0}-M_{1}\right)\rho_{c}\right]/M_{0}\rho_{w}\right\}\times 100\%$$

where *V_w_* is the pore volume of concrete, *Vc* is the volume of concrete, *ρ_c_* is the density of concrete, and *ρ_w_* is the density of water.

#### 2.4.3. Free Chloride Content

The free chloride content was measured every 12 drying–wetting cycles, up to 60 cycles. The concrete powder was collected every 3 mm at 0–30 mm away from the exposed surface. The collected drilling powders from each depth range were sieved by a 0.16 mm sieve, followed by oven drying under 60 °C for 12 h and then air cooling to 20 °C. According to Chinese standard JGJ/T 322-2013 [38], the free chloride content was determined using the DCCL-816 rapid chloride content tester.

#### 2.4.4. Chloride Ion Erosion Depth

The cut surface of the specimen was coated by 0.1 mol/L AgNO_3_ solution. After standing for 15 min, the area with chloride turned white due to AgCl. The depth of the white area was then measured with a vernier caliper at 10 mm intervals along the cross-section. The measured average value was the depth of chloride ion erosion of the specimen.

#### 2.4.5. Microstructure

The microstructure of concrete before and after drying–wetting cycles was observed by Regulus-8100 SEM. The samples with a size of 4 mm × 4 mm × 3 mm were selected from the concrete and dried in an oven at 60 °C for 24 h.

The phase of concrete before and after drying–wetting cycles was qualitatively analyzed by XRD. The concrete sample was ground into powder. Then, the powder was dried in an oven at 60 °C for 24 h. Data were collected using a D/MAX 2500 XRD with Cu Kα radiation at 40 mA and 40 kV. The 2theta angle range was set from 5° to 70°, and the scanning speed was 2°/min.

## 3. Results and Discussion

### 3.1. Compressive Strength

**Figure 3:**
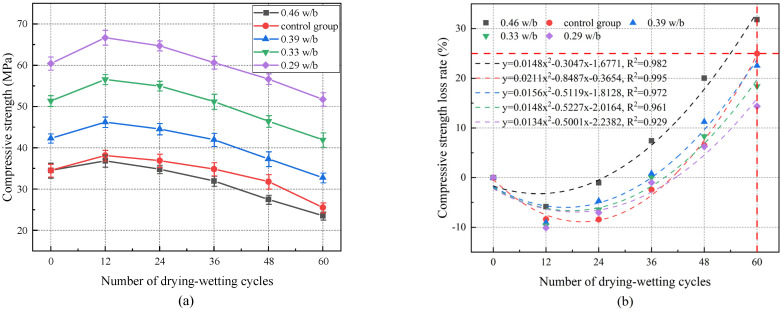
(**a**) Compressive strength of concrete with different w/b ratios under drying–wetting cycles. (**b**) Relationship between the number of drying–wetting cycles and compressive strength loss rate.

Figure 3a demonstrates that compressive strength of concrete with different w/b ratios increased first and then decreased with the increase in drying–wetting cycles. After 12 drying–wetting cycles, the maximum compressive strengths of 0.29, 0.33, 0.39, and 0.46 w/b were reached, which increased by 10.32%, 9.67%, 8.97%, and 5.85%, respectively, compared with the initial compressive strength. In the early stage of drying–wetting cycles, lower w/b led to a faster increase in compressive strength. This was because the lower w/b led to insufficient early hydration, and the remaining unhydrated cement particles underwent secondary hydration reactions during the drying–wetting cycle, which further filled the internal pores of the concrete and increased the strength. As the drying–wetting cycle continued, the stresses generated by dry shrinkage and wet expansion deformation formed a stress concentration at the weak interface between the internal structure of the concrete and the cement hydration products, leading to the formation of pores and cracks, and the damaged degree inside the concrete gradually increased [39,40]. At the same time, the above phenomena further provided space for the dry shrinkage and wet expansion of concrete, resulting in the rapid development of internal defects of concrete and ultimately a substantial decrease in strength. The compressive strength decreased at a greater rate with an increasing w/b in the later stage of the test. After 60 drying–wetting cycles, 0.46 w/b exhibited the highest compressive strength loss of (31.81%), whereas 0.29 w/b showed the smallest compressive strength loss (14.37%). It can be seen that concrete with lower w/b was more resistant to drying–wetting cycles than concrete with higher w/b. The relationship between the number of drying–wetting cycles and the compressive strength loss rate is depicted in Figure 3b. According to the fitting results, it is recommended that w/b should be less than 0.39 to ensure that the compressive strength of concrete meets the requirements under chloride drying and wetting environment.

Figure 3b shows that the compressive strength of 0.46 w/b was significantly lower than that of the control group. After 60 drying–wetting cycles, the compressive strength of 0.46 w/b decreased by 7.76% compared with the control group. This indicated that the dry shrinkage, wet expansion, and temperature stress caused by temperature changes in the variable temperature drying–wetting cycle test further promoted the expansion of internal pores and enhanced the damage weakening effect caused by the variable temperature drying–wetting cycles [23,24]. Therefore, the existing microcracks were expanded to form new cracks, and the strength was significantly reduced.

### 3.2. Splitting Tensile Strength

**Figure 4:**
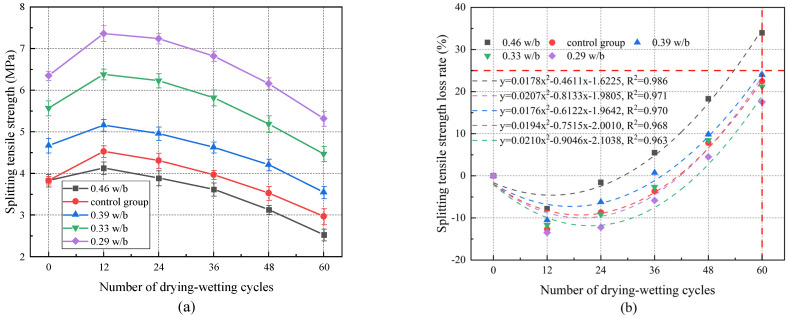
(**a**) Splitting tensile strength of concrete with different w/b ratios under drying–wetting cycles. (**b**) Relationship between the number of drying–wetting cycles and splitting tensile strength loss rate.

Figure 4a illustrates that the splitting tensile strength of concrete with different w/b ratios under drying–wetting cycles initially increased and subsequently decreased with the increase in drying–wetting cycles. The splitting tensile strengths of concrete with 0.29, 0.33, 0.39, and 0.46 w/b reached their peaks after 12 drying–wetting cycles, which increased by 14.87%, 13.24%, 14.77%, and 7.83%, respectively. This indicated that the smaller the w/b was, the more hydration products (AFt, Friedel’s) [41] were generated, and the denser the concrete was. The splitting tensile strength of concrete decreased gradually during 12 to 60 drying–wetting cycles. After 60 drying–wetting cycles, the splitting tensile strength of concrete with 0.29, 0.33, 0.39, and 0.46 w/b decreased by 16.22%, 19.74%, 23.98%, and 33.94%, respectively. The higher the w/b, the more severe the splitting tensile strength loss. The splitting tensile strength of 0.46 w/b was 14.81% lower than that of the control group after 60 drying–wetting cycles, which indicated that the change in temperature during the drying–wetting cycle test led to a significant reduction in the splitting tensile strength. The relationship between the number of drying–wetting cycles and the splitting tensile strength loss rate is depicted in Figure 4b. Based on the fitting results, when w/b was greater than 0.39, the splitting tensile strength loss rate exceeded 25%. It is suggested that w/b should be 0.29~0.39 to ensure the safety and reliability of concrete structures under drying and wetting corrosion of chloride salt.

The variation law of splitting tensile strength of concrete is consistent with that of compressive strength, that is, it increases first and then decreases. The intrinsic reason for the change in the splitting tensile strength of concrete during the drying–wetting cycle test is the same as that of compressive strength. In the early stage of corrosion, the chemical reaction within the concrete fills the internal pores, and the microstructure is denser, resulting in a corresponding increase in strength. As the test progresses to the later stage, the adverse effects of drying–wetting cycles on concrete gradually dominate, making the cracks inside concrete develop and the strength decrease. In addition, the increase in temperature during the drying–wetting cycles exacerbates the development of concrete cracks, thus intensifying the adverse effects on its mechanical properties [42,43].

### 3.3. Mass Loss Rate

**Figure 5:**
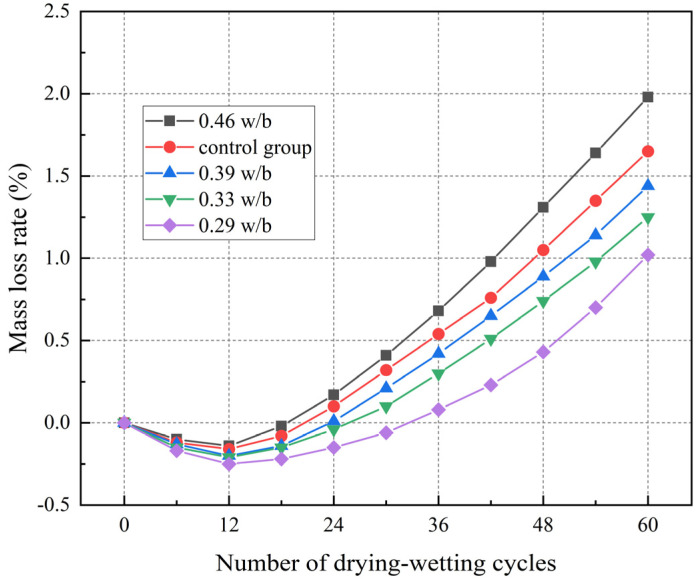
Mass loss rate of concrete with different w/b ratios under drying–wetting cycles.

Figure 5 shows the variation of the mass loss rate of concrete with different w/b ratios during drying–wetting cycles. It can be concluded that the mass of concrete initially increased and then decreased with the increase in drying–wetting cycles. The peaks of concrete with 0.29, 0.33, 0.39, and 0.46 w/b increased by 0.25%, 0.21%, 0.19%, and 0.14%, respectively. The increase in mass was attributed to the following: (1) The secondary hydration process of chloride ions and unhydrated cement particles was longer after the entry of water during the drying–wetting cycle, the reaction products filled the internal pores of the concrete, the concrete was denser, and the lower w/b led to a faster mass growth rate. (2) In the drying phase, water escaped and evaporated, the concentration of salt solution in the pores increased, the crystallization of chloride salt was attached to the surface of the specimen, and the mass increased [44,45].

With the continuous drying–wetting cycles, the expansion stresses generated by salt crystallization products and expansion products coarsened the pores. Finally, the internal pore structure of concrete was destroyed, the mortar fell off, and the internal particles dissolved, leading to a rapid decline in the mass of concrete. After 60 drying–wetting cycles, 0.46 w/b exhibited the highest mass loss (1.98%), whereas 0.29 w/b showed the smallest mass loss (1.02%). Compared with the control group, the mass of 0.46 w/b decreased by 0.33%. Therefore, the increase in temperature further aggravated the above phenomenon, accelerates mortar falling off, and the loss of concrete mass was more serious [23].

### 3.4. RDEM

**Figure 6:**
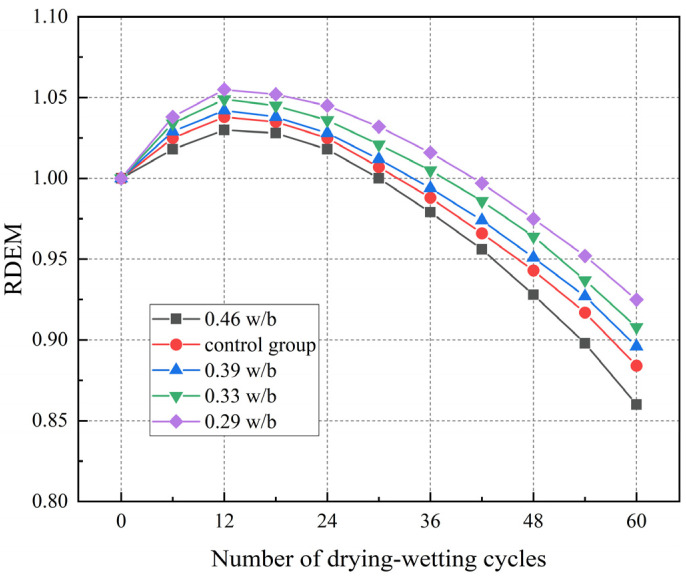
RDEM of concrete with different w/b ratios under drying–wetting cycles.

It can be seen from Figure 6 that the variation law of RDEM under different w/b ratios initially increased before declining. In the initial stage of drying–wetting cycles, the RDEM of concrete specimens increased slightly, and the RDEM peaks with 0.29, 0.33, 0.39, and 0.46 w/b were 105.51%, 104.90%, 104.20%, and 103.10% of the initial values, respectively. These peaks were due to the reaction of chloride ions with Ca(OH)_2_ and AFm to form corrosion products (AFt, Friedel’s), which filled the internal pores and reduced the porosity, thereby improving the RDEM [46].

With the increase in drying–wetting cycles, the RDEM of concrete began to decrease. After 60 drying–wetting cycles, the RDEM of 0.46 w/b decreased the most (14.00%), while those of 0.29, 0.33, and 0.39 w/b decreased by 7.50%, 9.20%, and 10.40%, respectively. The internal structure of concrete with lower w/b was dense, and the ability to resist drying–wetting cycle corrosion was better than that of concrete with higher w/b. The pore structure of concrete with higher w/b was destroyed under the action of drying–wetting cycles, which led to the continuous generation and expansion of microcracks, finally forming a continuous pore structure, and the porosity became larger. Compared with the control group, the RDEM of 0.46 w/b decreased faster. These results indicated that the deterioration degree of RDEM was intensified by a higher w/b and temperature.

### 3.5. Porosity

**Figure 7:**
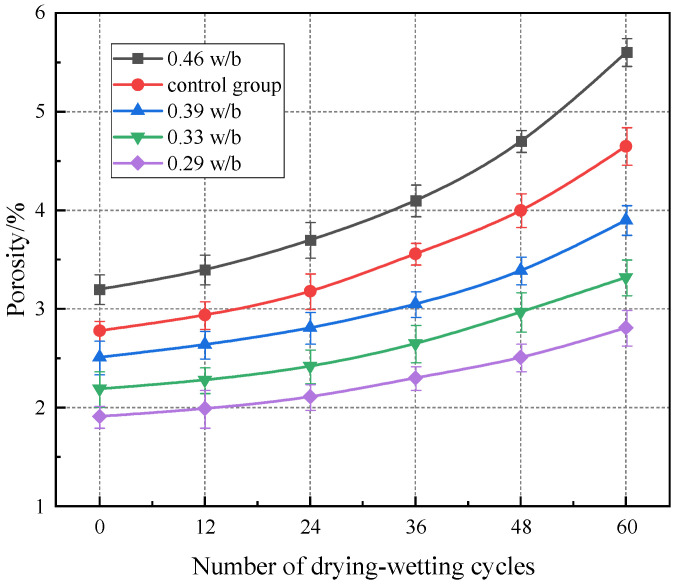
Porosity of concrete with different w/b ratios under drying–wetting cycles.

The variation of concrete porosity with the number of drying–wetting cycles is shown in Figure 7. As shown in Figure 7, the porosity of concrete increased exponentially with drying–wetting cycles increasing. In the early stage of the test (0–24 cycles), the porosity increased slowly, and in the later stage of the test (24–60 cycles), the rate of porosity increase became faster. After 60 drying–wetting cycles, the porosities of 0.29, 0.33, 0.39, and 0.46 w/b increased by 47.12%, 51.60%, 55.38%, and 75.00%, respectively. The porosity of concrete increased continuously with w/b increasing. After 60 drying–wetting cycles, the porosities of 0.33, 0.39, and 0.46 w/b increased by 18.15%, 38.79%, and 99.29%, respectively, compared with 0.29 w/b. The increase in the porosity of concrete with higher w/b was more obvious under the action of drying–wetting cycles.

During the drying process, the gas escaped outward, the internal pores of the concrete were further expanded, and even new pores and cracks were generated. In the wetting process, water entered the internal pores of concrete under the action of capillary pressure, causing pore expansion and further expansion of cracks and finally forming a continuously connected pore structure [44,47]. The porosity of concrete with 0.46 w/b was reduced by 20.43% compared with the control group. The temperature cycling test resulted in the rapid development of concrete pores and decreased the porosity.

### 3.6. Free Chloride Content

**Figure 8:**
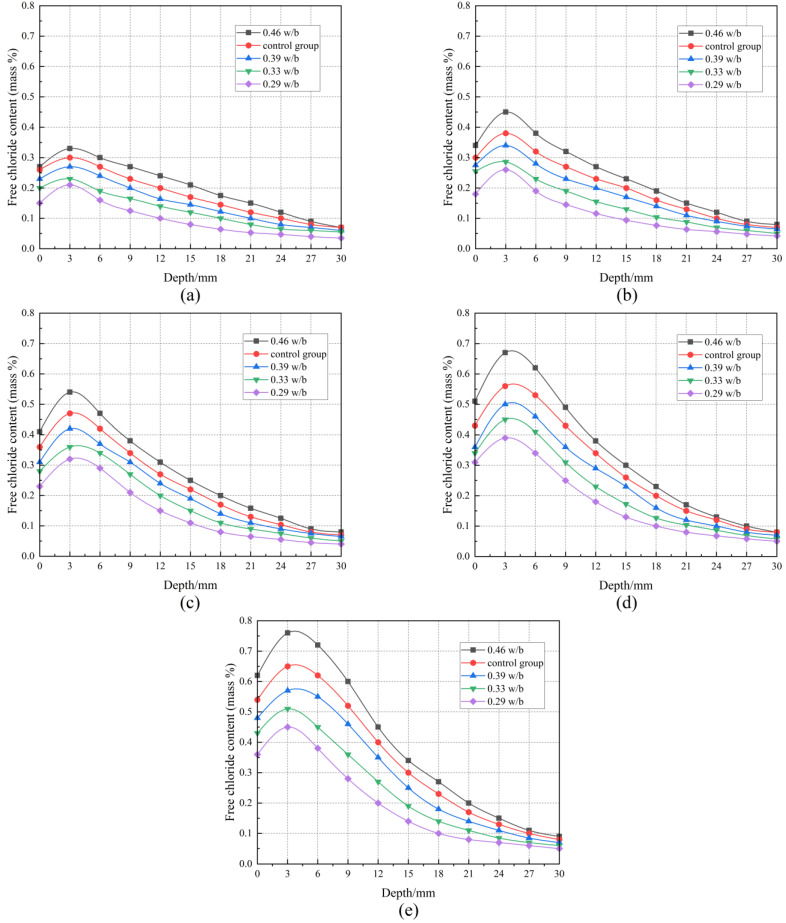
The distribution of free chloride content after different drying–wetting cycles: (**a**) 12 cycles, (**b**) 24 cycles, (**c**) 36 cycles, (**d**) 48 cycles, and (**e**) 60 cycles.

**Figure 9:**
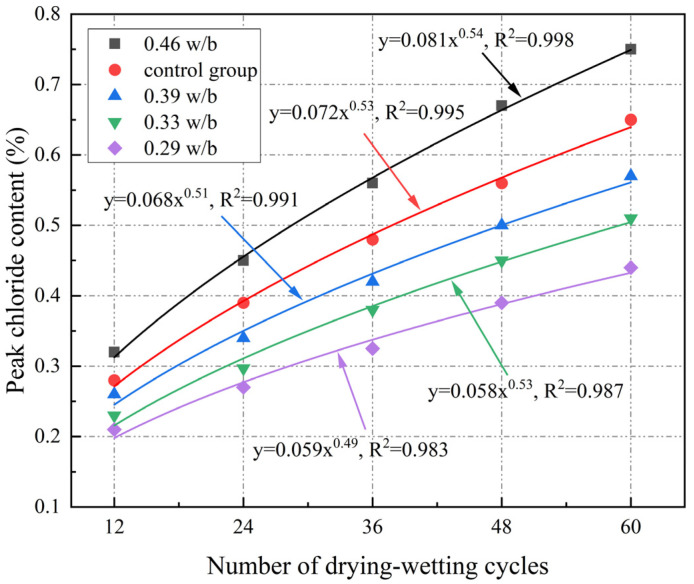
Fitting curve of peak chloride concentration.

It can be seen from Figure 8 that the free chloride content of concrete initially increased and then decreased with the increase in the erosion depth under different drying–wetting cycles. At the same erosion depth, the free chloride content with different w/b ratios increased with drying–wetting cycles increasing, but the increase in the free chloride content of 0.46 w/b was greater than that of 0.29 w/b, which indicated that concrete with higher w/b had worse resistance to chloride corrosion under drying–wetting cycles. As can be seen in Figure 8, at 0–3 mm, free chloride content gradually increased with the increase in the erosion depth due to the coupling of convection and diffusion, and free chloride content peaked at 3 mm. At 3–21 mm, free chloride content decreased with erosion depth increasing, which was attributed to convection limiting the transport of chloride ions in deeper layers of concrete, which were mainly transported by diffusion. At 21–30 mm, free chloride content eventually stabilized with erosion depth increasing. The convection zone depth of concrete was approximately 3 mm in the drying–wetting cycle mechanism, which was consistent with Cao et al. [48]. Figure 9 illustrates that peak chloride content increased as a power function with increasing drying–wetting cycles, and the higher the w/b, the better the correlation of the fitting results.

According to Figure 9, the peak free chloride content of 0.46 w/b was 0.76%, which was 70.45%, 47.06%, and 31.58% higher than that of 0.29, 0.33, and 0.39 w/b, respectively. This was because continuous drying–wetting alternation accelerated crack expansion, and connectivity, and eventually became fast channels for chloride ion transport, resulting in more chloride ions penetrating into the concrete interior under the same drying–wetting cycles [49]. An increase in w/b reduced the chloride corrosion resistance of concrete, and this effect became more pronounced with the increase in w/b. This also confirmed that the chloride ion erosion depth increased with w/b increasing.

After 12 and 60 drying–wetting cycles, the differences in the free chloride content of concrete with different w/b ratios at different depths are listed in Table 6. Table 6 shows that the free chloride content of 0.46 w/b at different depths was greater than that of 0.29 w/b, and this increasing trend was more obvious with the increase in drying–wetting cycles. After 60 drying–wetting cycles, the free chloride content of 0.46 w/b at 3 mm increased by 0.43% compared with 12 drying–wetting cycles, while that of 0.29 w/b increased by only 0.24%. The smaller the w/b, the greater the difference in the free chloride content, indicating that the concrete surface suffered more severe chloride salt corrosion. Compared with the control group, the difference in free chloride content of 0.46 w/b at 0–12 mm depth was larger, indicating that temperature further accelerated the diffusion rate and penetration depth of chloride ions, reducing the chloride resistance of concrete.

### 3.7. Chloride Ion Erosion Depth

**Figure 10:**
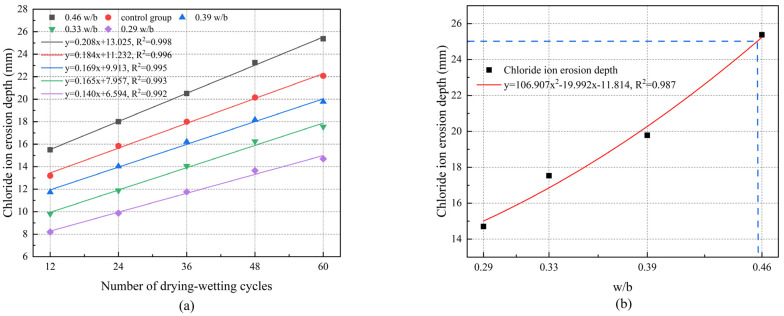
(**a**) Chloride ion erosion depth of concrete under different drying–wetting cycles. (**b**) Relationship between w/b and chloride ion erosion depth.

As shown in Figure 10a, the chloride ion erosion depth increased with the increase in drying–wetting cycles, and the chloride ion erosion depth was deeper at higher w/b. After 60 drying–wetting cycles, the erosion depths of 0.29, 0.33, 0.39, and 0.46 w/b were 14.70 mm, 17.54 mm, 22.08 mm, and 25.38 mm, respectively. The chloride ion erosion depth of 0.46 w/b was 72.65%, 44.70%, and 14.95% higher than that of 0.29, 0.33, and 0.39 w/b, respectively. This was because repeated drying–wetting cycles produced a higher concentration gradient difference and stronger capillary force, so the deterioration of internal pores and microcracks of concrete with higher w/b further increased the formation of continuous pores, which provided more channels for chloride ion transmission, thereby accelerating the diffusion rate of chloride ions, and the depth of chloride ion erosion spread more deeply [12,43]. The concrete with lower w/b had fewer internal defects and higher internal structure compactness, which could better resist chloride ion corrosion to the interior, so the erosion depth was smaller. The relationship between w/b and the chloride ion erosion depth is depicted in Figure 10b. According to the fitting results, the chloride ion erosion depth of w/b = 0.46 was more than 25 mm, which exceeded the minimum protective layer thickness of the concrete structure under drying–wetting alternate environments. It is recommended that w/b should be less than 0.39 to ensure that concrete has excellent resistance to chloride corrosion.

Additionally, under the same drying–wetting cycles, the chloride ion erosion depth of 0.46 w/b was greater than that of the control group. After 60 drying–wetting cycles, the chloride ion erosion depth of 0.46 w/b was 14.95% higher than that of the control group. This could be attributed to the effect of the variable test temperature on chloride ion transport. The increase in the temperature accelerated the reaction rate and diffusion rate of chloride ions [24].

### 3.8. Microstructure

#### 3.8.1. SEM

**Figure 11:**
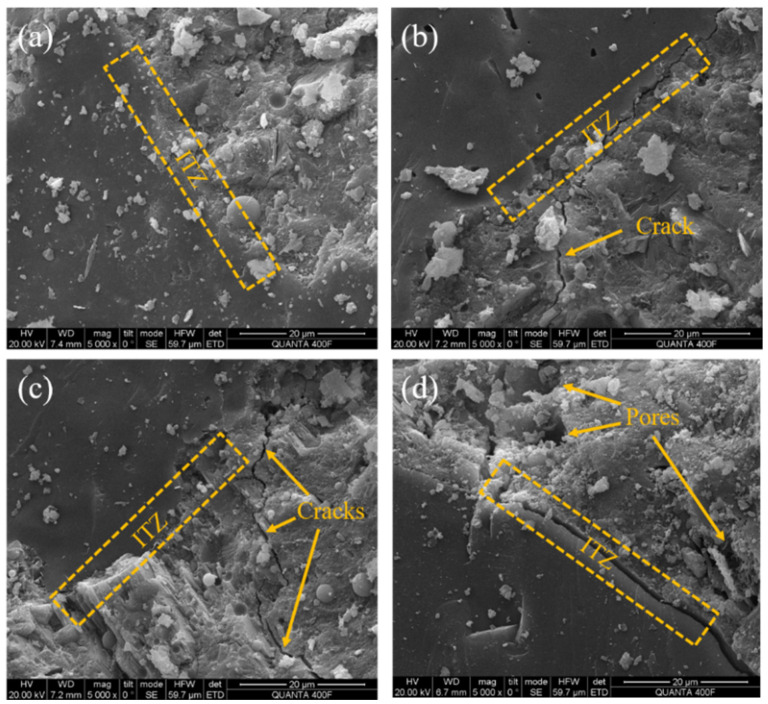
SEM images of ITZ before drying–wetting cycles: (**a**) 0.29 w/b, (**b**) 0.33 w/b, (**c**) 0.39 w/b, and (**d**) 0.46 w/b.

The microstructure of concrete with different w/b ratios before drying–wetting cycles is shown in Figure 11. In Figure 11a, the internal particles are closely connected, the interface transition zone (ITZ) is smooth and clear, and the aggregate is closely bonded to the mortar without pores and cracks. Figure 11b,c have smooth and dense surfaces with a few cracks appearing. Figure 11d has a large number of holes, cracks become wider, and the bonding at the interface is weak. It can be seen that the lower w/b has a denser internal structure with lower porosity.

**Figure 12:**
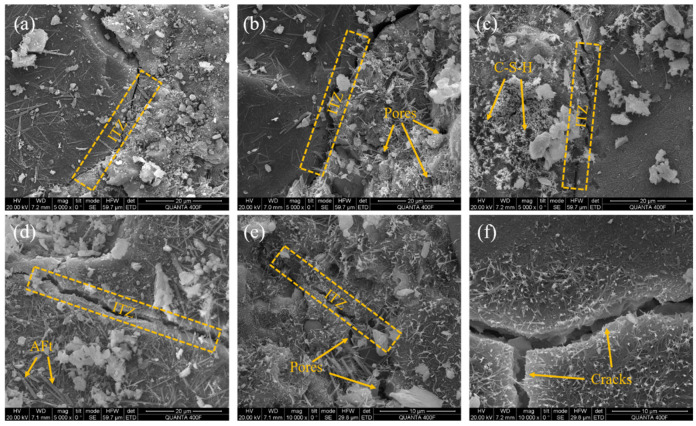
SEM images of ITZ after 60 drying–wetting cycles: (**a**) 0.29 w/b, (**b**) 0.33 w/b, (**c**) 0.39 w/b, (**d**) control group, (**e**) 0.46 w/b, and (**f**) cracks.

The microstructure of concrete with different w/b ratios after 60 drying–wetting cycles is depicted in Figure 12. Compared with Figure 12a, the microstructure of concrete in Figure 12b becomes looser, accompanied by the formation of more cracks and pores. This shows that the increase in w/b aggravated the internal damage of concrete and widened the width of the ITZ. As shown in Figure 12c,d, abundant AFt and C-S-H can be observed near the cracks, and AFt grows extensively in the connected pores, which can easily cause local stress concentration, leading to deterioration of the pore structure. Figure 12e has the loosest structure, a large number of chloride products are distributed on the surface, cracks and pores are increased, and the interface is disordered. These cracks weakened the binding capacity of the aggregate and mortar and provided more channels for chloride ion penetration. As shown in Figure 12a–e, chloride ions reacted with C_3_A and AFm to form Friedel’s, and the OH− in the interlayer of AFm hydrates reacted with free chloride ions in the pore solution to form Friedel’s. The continuous occurrence of chemical reactions weakened the microstructure. Additionally, repeated temperature cycles led to a greater chloride gradient difference and stronger capillary force, which further accelerated the diffusion of the chloride ions, resulting in the interpenetration of cracks and pores and the increasing crack width [28,50], as shown in Figure 12f. The above phenomenon was further aggravated with the increase in w/b and temperature and eventually led to the reduction in chloride resistance of concrete.

Microstructural difference was the fundamental reason for the change in macro performance. After 60 drying–wetting cycles, the microstructure of concrete with 0.29 w/b was denser, and the bonding performance between aggregate and mortar was better. The compressive strength was higher, and the chloride resistance was better. However, the microstructure of concrete with 0.46 w/b was looser, and pores and cracks expanded. The compressive strength decreased, and the resistance to chloride corrosion became worse. Compared with constant temperature tests, variable temperature drying–wetting cycles caused more cumulative damage. This was because the increase in temperature accelerated the diffusion rate and penetration depth of chloride ions, increased the solubility of corrosion products and hydration products, and led to pore expansion and crack propagation [51]. The mechanical properties and chloride resistance of concrete continue to decline.

#### 3.8.2. XRD

**Figure 13:**
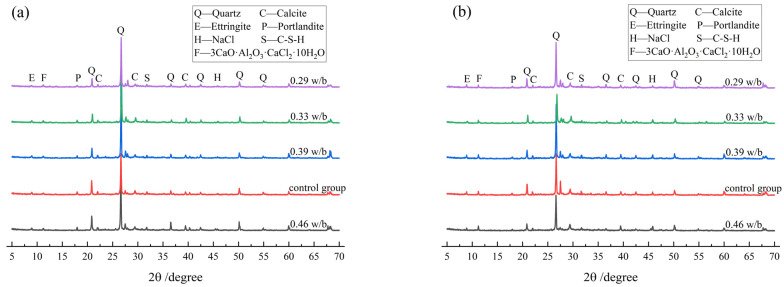
XRD patterns of concrete with different w/b ratios: (**a**) before drying–wetting cycles and (**b**) after 60 drying–wetting cycles.

Figure 13 shows the XRD diffraction patterns of concrete with different w/b ratios before and after drying–wetting cycles. As can be seen from Figure 13, the main hydration products of concrete with different w/b ratios before and after drying–wetting cycles were Ca(OH)_2_, Aft, and C-S-H gel. Compared with concrete with larger w/b, the Ca(OH)_2_ diffraction peak of concrete with smaller w/b gradually weakened, and the Ca(OH)_2_ diffraction peak at 17.63° almost disappeared after 60 drying–wetting cycles, which was attributed to the fact that the secondary hydration reaction of concrete with lower w/b consumed Ca(OH)_2_ and promoted the formation of C-S-H gel and Friedel’s. The diffraction peak of AFt appeared at 8.89°, and the diffraction peak of AFt increased with the increase in drying–wetting cycles. This indicated that a longer wetting time was favorable for the chemical reaction to generate more AFt, which led to a denser microstructure. Additionally, the diffraction peak of CaCO_3_ was observed at 29.36°, which was due to the carbonation reaction during the drying process.

As shown in Figure 13, the diffraction peak of Friedel’s appeared at 11.48°, and the intensity of Friedel’s diffraction peak with 0.46 w/b concrete was higher than that of the control group, which indicated that the variable temperature drying–wetting cycle accelerated the reaction rate and diffusion rate of chloride ions in concrete and further promoted the formation of Friedel’s. The diffraction peak of NaCl appeared at 46.21°, which also indicated that there were more chloride products distributed in the SEM in Figure 12.

## 4. Conclusions

(1) With the increase in drying–wetting cycles, the performance of concrete under different w/b ratios initially increased before declining. After 60 drying–wetting cycles, the concrete with 0.46 w/b deteriorated the most, and its compressive strength, splitting tensile strength, mass, and RDEM decreased by 31.81%, 33.94%, 1.98%, and 14.00%, respectively.

(2) Higher w/b increased porosity, chloride diffusion depth, and chloride content, thus reducing resistance to chloride corrosion. After 60 drying–wetting cycles, the compressive strength, splitting tensile strength, mass, and RDEM of concrete with 0.46 w/b decreased by 54.50%, 52.44%, 0.96%, and 6.5%, respectively, as compared with that of concrete with 0.29 w/b. Higher w/b was more likely to increase the chloride penetration and erosion depth within identical drying–wetting cycle periods. After 60 drying–wetting cycles, compared with concrete with 0.29 w/b, 0.33 w/b, and 0.39 w/b, the peak chloride content of concrete with 0.46 w/b increased by 70.45%, 47.06%, and 31.58%, respectively, and the erosion depth increased by 72.65%, 44.70%, and 14.95%.

(3) Compared with the drying–wetting cycle with a constant temperature, the deterioration of the concrete performance was more serious under the drying–wetting cycle with variable temperature. The increase in the test temperature significantly accelerated the diffusion rate, penetration depth, and chemical binding capacity of chloride ions. Compared with the control group, the compressive strength, splitting tensile strength, mass, and RDEM of concrete with 0.46 w/b decreased by 7.76%, 14.81%, 0.33%, and 2.41%, respectively. The peak chloride content and chloride ion erosion depth of concrete with 0.46 w/b were 15.38% and 14.95% higher than those of control group after 60 drying–wetting cycles.

(4) Microscopic analysis revealed that a higher w/b under the variable temperature drying–wetting cycle accelerated the deterioration of the concrete microstructure, which was the fundamental reason for the decay of the mechanical properties and the decline in the chloride corrosion resistance. The higher w/b accelerated the expansion and connection of pores and cracks, increasing the porosity, and led to a looser microstructure. The variable temperature cycle exacerbated the internal damage of concrete, widened the ITZ width, and decreased the interfacial bonding properties. According to the numerical fitting analysis, the w/b should be 0.29~0.39 under the condition that the mechanical properties and chloride corrosion resistance of concrete are met.

## Figures and Tables

**Table 1:** The chemical compositions of cement, fly ash, and silica fume.

Components	Cement	Fly Ash	Silica Fume
Chemical composition (%)			
SiO_2_	22.16	43.86	95.4
Al_2_O_3_	6.67	31.23	0.84
Fe_2_O_3_	4.23	9.18	0.18
CaO	60.13	9.11	1.96
MgO	1.33	1.04	0.75
SO_3_	1.76	0.75	–
TiO_2_	0.23	1.14	–
K_2_O	0.42	2.07	–
Loss on ignition	2.56	1.87	1.79

Note: “–” is not measured items.

**Table 2:** The main properties of aggregate.

Aggregate Type	Apparent Density/ kg/m^3^	Bulk Density/ kg/m^3^	Water Absorption/ %	Crush Value/ %
NCA	2718	1576	0.7	4.2
NFA	2636	1549	1.3	8.7

**Table 3:** Mix proportions of concrete (kg/m^3^).

Mixture	w/b	NCA	NFA	Cement	FLY ASH	Silica Fume	Water	SP
Control group	0.46	1084.42	661.69	289.65	57.93	38.62	180.86	1.93
A	0.46	1084.42	661.69	289.65	57.93	38.62	180.86	1.93
B	0.39	1121.86	625.38	320.54	64.11	42.74	167.08	2.14
C	0.33	1158.86	589.50	351.07	70.21	46.81	153.47	2.34
D	0.29	1183.90	565.21	371.74	74.35	49.56	144.26	2.48

**Table 4:** Average test temperatures corresponding to different months.

Months	Mean Temperature/°C	Test Temperature/°C
1, 2, 3, 11, 12	4.2	24
4, 10	12.4	35
5, 6, 9	21.2	43
7, 8	28.0	49

**Table 5:** Water temperatures of drying–wetting cycle.

Drying–Wetting Cycle	1	2	3	4	5	6	7	8	9	10	11	12
Temperature/°C	24	24	24	35	43	43	49	49	43	35	24	24

**Table 6:** Difference of free chloride content at different depths after 12 and 60 drying–wetting cycles.

Depth (mm)	Δ(%)										
	0	3	6	9	12	15	18	21	24	27	30
0.46 w/b	0.35	0.43	0.42	0.33	0.21	0.13	0.10	0.05	0.03	0.02	0.02
Control group	0.28	0.35	0.34	0.29	0.20	0.12	0.09	0.04	0.03	0.02	0.01
0.39 w/b	0.25	0.30	0.29	0.26	0.18	0.11	0.08	0.04	0.03	0.02	0.01
0.33 w/b	0.23	0.28	0.26	0.20	0.13	0.07	0.06	0.04	0.03	0.02	0.01
0.29 w/b	0.21	0.24	0.22	0.16	0.10	0.06	0.05	0.03	0.02	0.01	0.01

## Data Availability

The raw data supporting the conclusions of this article will be made available by the authors on request.
